# 编者按

**DOI:** 10.3724/SP.J.1123.2021.08025

**Published:** 2021-10-08

**Authors:** Editorial Office of Chinese Journal of Chromatography

张玉奎,中国科学院院士,河北省保定市人。1942年出生,1965年毕业于南开大学化学系,1965年至今在中国科学院大连化学物理研究所工作,已从事分离科学事业近六十载。张玉奎院士主要从事色谱基本理论和新技术、新方法的研究。他勤于耕耘,硕果累累,为我国色谱学科的发展做出了巨大的贡献。

张玉奎院士在基础研究方面,建立了系统的色谱热力学和动力学研究方法,为液相色谱专家系统的建立奠定了理论基础;在建立液相色谱专家系统总体布局过程中,提出了“知识库必须基于色谱理论和经验规律相结合”的思想,并以大量的事实验证了知识库的正确性和可靠性。他在毛细管电泳的理论研究中,发现了毛细管区带电泳中操作参数及溶质的分子结构对迁移行为的影响规律;同时发展了物理吸附涂层、混合填料、整体聚合床层等多种电色谱柱系统;系统研究了溶质在不同柱系统上的分离机制和富集规律。

在深入理论研究的基础上,张玉奎院士特别注重完成国家任务与仪器的应用开发。承担了“密闭舱大气组分分析仪”、2030型智能高效液相色谱仪样机、高压输液泵、紫外可变波长检测器等整套液相色谱仪器产品、k-1吸附型高效液相色谱柱、1~5 mm内径的吸附型高效液相色谱柱、生物工程下游目标产品分离纯化用径向色谱柱等研制工作。大部分产品已实现产业化,并荣获多项科技成果奖。

近年来,结合国家重大需求,张玉奎院士放眼于分析学科与生命学科的交叉融合发展,带领研究团队开展蛋白质组定性定量、结构和相互作用分析新方法研究,显著提高了蛋白质组分析的覆盖度、精准度和分析通量。目前上述新方法已用于解决细胞生物学、精准医学、合成生物学、生物制药、公共安全等领域的瓶颈问题。

张玉奎院士的科研工作与我国色谱事业共同发展。上述不同时期的基础和应用研究成果反映出我国色谱学科体系的发展与创新过程,不仅为当前复杂体系分析研究提供了重要的理论支撑,而且对于分离分析方法与生命科学的交叉融合具有指导价值。张玉奎院士著述颇丰,在国内外发表论文500余篇,撰写专著7部,授权120项发明专利。他还积极参加各种学术活动,为学科建设争取各方的理解和支持,积极推动我国色谱学科蓬勃发展,不断进步,位列国际先进水平。

张玉奎院士还特别重视学术出版,多年来投入大量心血指导《色谱》期刊的发展。他自1996年担任《色谱》主编至今,始终坚持服务中国色谱学科的初心,团结一流专家学者,挖掘青年人才,指导期刊重点跟踪和报道我国的学术理论和科技成果,面向国家重点、难点问题和任务,并不断挖掘新的科学增长点,不断探索将交叉学科纳入发表范围,推动色谱等分离手段在诸多应用领域发挥越来越重要的作用。在张玉奎院士的悉心指导之下,《色谱》期刊努力学习国内外先进的办刊经验,提升出版质量,加快出版速度,推出一系列颇受好评的专辑和文章。获得中国精品科技期刊等荣誉称号,由中国科学技术信息研究所发布的印证指标一直名列前茅。

张玉奎院士虽八十高龄,仍一直工作在科研的第一线,治学不辍,诲人不倦。他坚持以人为本的原则,高度重视人才培养,可谓桃李满天下。他为分析科学领域培养的大批人才,有的已经成为博士生导师,还有的在仪器公司成为科研骨干;在基础研究、应用开发以及仪器产业化等方面发挥着重要的作用。先生锲而不舍、锐意进取、扎实严谨、爱岗敬业的科学家精神,广受爱戴和敬仰,是大家学习的楷模和典范。

值此张玉奎院士八十华诞之际,中国科学院大连化学物理研究所张丽华研究员担任客座主编,约请诸多专家撰文集结成专辑。该专辑在一定程度上反映了张玉奎院士多年来的研究兴趣和当今前沿进展,衷心感谢不辞辛劳组织约稿的张丽华研究员,以及各位论文作者和审稿专家的大力支持。借此机会,谨祝愿先生福如东海,寿比南山,吉祥如意,身体健康!

